# Regulation of COX Assembly and Function by Twin CX_9_C Proteins—Implications for Human Disease

**DOI:** 10.3390/cells10020197

**Published:** 2021-01-20

**Authors:** Stephanie Gladyck, Siddhesh Aras, Maik Hüttemann, Lawrence I. Grossman

**Affiliations:** 1 Center for Molecular Medicine and Genetics, Wayne State University School of Medicine, Detroit, MI 48201, USA; sgladyck@med.wayne.edu (S.G.); saras@wayne.edu (S.A.); mhuttema@med.wayne.edu (M.H.); 2 Perinatology Research Branch, Division of Obstetrics and Maternal-Fetal Medicine, Division of Intramural Research, Eunice Kennedy Shriver National Institute of Child Health and Human Development, National Institutes of Health, U.S. Department of Health and Human Services, Bethesda, Maryland and Detroit, MI 48201, USA

**Keywords:** intermembrane space proteins, ETC complex assembly, mitochondrial regulation

## Abstract

Oxidative phosphorylation is a tightly regulated process in mammals that takes place in and across the inner mitochondrial membrane and consists of the electron transport chain and ATP synthase. Complex IV, or cytochrome *c* oxidase (COX), is the terminal enzyme of the electron transport chain, responsible for accepting electrons from cytochrome *c*, pumping protons to contribute to the gradient utilized by ATP synthase to produce ATP, and reducing oxygen to water. As such, COX is tightly regulated through numerous mechanisms including protein–protein interactions. The twin CX_9_C family of proteins has recently been shown to be involved in COX regulation by assisting with complex assembly, biogenesis, and activity. The twin CX_9_C motif allows for the import of these proteins into the intermembrane space of the mitochondria using the redox import machinery of Mia40/CHCHD4. Studies have shown that knockdown of the proteins discussed in this review results in decreased or completely deficient aerobic respiration in experimental models ranging from yeast to human cells, as the proteins are conserved across species. This article highlights and discusses the importance of COX regulation by twin CX_9_C proteins in the mitochondria via COX assembly and control of its activity through protein–protein interactions, which is further modulated by cell signaling pathways. Interestingly, select members of the CX_9_C protein family, including MNRR1 and CHCHD10, show a novel feature in that they not only localize to the mitochondria but also to the nucleus, where they mediate oxygen- and stress-induced transcriptional regulation, opening a new view of mitochondrial-nuclear crosstalk and its involvement in human disease.

## 1. Introduction

Mitochondria are the major source of cellular energy that is required to sustain life. They are double-membrane organelles in which the process of cellular respiration and ATP production takes place. This process, oxidative phosphorylation, occurs at the electron transport chain (ETC), a series of four protein complexes embedded in the inner mitochondrial membrane (IM). The complexes create a proton gradient by pumping protons from the matrix to the intermembrane space (IMS), which is coupled with electron transfer down the chain. The electrochemical proton gradient thereby produced is used by ATP synthase (complex V) to generate ATP from ADP and phosphate. 

Complex IV, or cytochrome *c* oxidase (COX), is the terminal enzyme of the ETC and is responsible for reducing oxygen to water. Physiologically, the mammalian complex is a dimer, with each monomer composed of 13 tightly bound subunits embedded in the IM, an assembly supported by several crystal structures resolved from COX in bovine heart [1,2]. However, more recently, monomeric crystal structures of COX were also published [3,4] and monomeric COX was also reported in a supercomplex [5]. It is therefore possible that an equilibrium exists between dimeric and monomeric COX, which could be subject to regulation. In addition, a 14th subunit has been proposed—NDUFA4—which was originally believed to be a subunit of complex I [6,7]. A structural study showed that NDUFA4 appears to be a subunit in the COX monomer, likely adding to the stability of the complex [7]. NDUFA4 as part of the COX monomer is located at the interface of the dimeric complex, where it would prevent or interfere with dimer formation and which could be a reason that the protein was never detected in the dimeric crystal structure. The validity of NDUFA4′s role as a true subunit has been questioned and it was argued that, because NDUFA4 may bind to both complexes I and IV and is not consistently found in COX preparations, it may function as an assembly factor for the respirasome [8]. 

The three largest subunits are encoded by the mitochondrial genome whereas the other subunits are encoded by the nuclear genome. Among the mitochondrial-encoded subunits, subunits I and II contain the catalytic centers. The latter consist of metal centers that are involved in the electron acceptance from complex III via cytochrome *c* and the pathway of the electron through the complex itself: electrons received from cytochrome *c* first reach the Cu_A_ center in subunit II, are then transferred to heme *a* in subunit I, and finally reach the heme *a_3_*-Cu_B_ site of subunit I, where oxygen is reduced to water. 

There are various modes of regulation of COX activity [1], summarized in Table 1. The purpose of this review is to explore the regulation of COX through the interaction with proteins of the twin CX_9_C family. Members of this protein family have been shown to be important in COX complex assembly and function, as well as for direct regulation of the oxidase [9] (Table 2). Note that the 13 tightly bound COX subunits are traditionally distinguished by Roman numerals introduced by the Kadenbach lab, whereas auxiliary proteins are designated with Arabic numerals (yeast nomenclature can be found in Table 2).

The twin CX_9_C family of proteins is characterized by its unique motif of two cysteines separated by usually nine amino acid residues. This motif is found in the coiled-coil-helix-coiled-coil-helix (CHCH) domain, where pairs of cysteines form a helix turn helix fold by forming disulfide bonds with one another [10,11,12]. Another family of proteins, called the “small Tim” proteins, contains a similar but shorter twin CX_3_C motif and plays chaperone roles in the TIM22 pathway for insertion of proteins into the IMS-facing side of the inner membrane (IM) [13]. The CHCH domain is important for the import of the proteins into the intermembrane space (IMS) of the mitochondria. IMS import is facilitated through the Mia40/CHCHD4 redox mechanism [14,15]. The first studies of this family of proteins took place in *Saccharomyces cerevisiae*, where a detailed study found that 13 of the 14 yeast family members were conserved across species [16]. A follow-up study contained a genome-wide analysis to determine family member functions, with six of the CX_9_C proteins determined to be involved in COX assembly [9]. Recently, more information has become available through further research into the function of twin CX_9_C family members.

## 2. COX Regulation through Assembly

**Figure 1:**
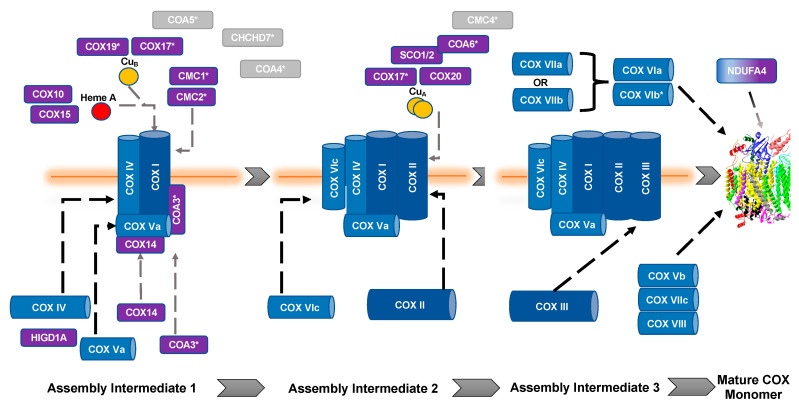
Assembly of cytochrome *c* oxidase. The assembly of COX is thought to be both modular and linear. Dark blue, mitochondrial encoded COX core subunits; light blue, nuclear encoded COX subunit; purple, assembly factors; gray, unknown function. *, Twin CX_9_C protein. Gray dashed lines represent assembly factor interactions. Black dashed lines represent subunit assembly.

The biogenesis and maturation of COX is critical for its proper function. There are multiple steps in this tightly regulated process: the insertion of metal groups in COX I and COX II, the import and folding of nuclear encoded subunits, and the proper assembly of the subunits into the complex. Over 30 auxiliary proteins are involved in the biogenesis of the core enzyme composed of COX I, COX II, and COX III [17]. The hypothesized assembly pathway favors a modular–linear assembly, where subunits are first assembled into module intermediates and then these modules are assembled into the COX monomer (Figure 1) [18,19]. The first step of monomer assembly is the synthesis of mitochondrially encoded COX I, including the insertion of heme *a*, which is followed by its association with the COX IV and COX Va module [18,20]. The COX II module, which requires the insertion of the Cu_A_ center into the subunit before assembly can continue [21,22,23,24], forms a complex intermediate with COX VIc, COX VIIb, COX VIIc, and COX VIIIa. The COX III module consists of COX III, COX VIa, COX VIb, and COX VIIa. These modules are then assembled in a linear fashion, upon which NDUFA4 interacts to assist in the stabilization of the COX monomer [18]. Detailed COX biogenesis and assembly has been reviewed elsewhere [19]; however, summaries of assembly will be provided where necessary.

It is important to note that copper metabolism and homeostasis in the mitochondria are important in aerobic respiration of the cell (for detailed reviews, see [25,26]). All of the assembly proteins discussed in this section have been shown to be involved or associated with copper transport and moiety insertion into appropriate subunits during COX assembly, which is an absolute necessity for the complex to function. Chaperones are very important in this process because of the redox sensitivity of the transition metal that, if left unchecked, can become a source of ROS (for review, see [27]).

Most of the twin CX_9_C proteins involved in COX assembly were first identified in yeast in a screen to identify proteins that affect OXPHOS; the proteins that were shown to affect OXPHOS were COX17, COX23, COX19, CMC1, and CMC2 [16]. COA4 and COA6 were identified in separate studies [28,29].

### 2.1. COX Subunit I Module Assembly

COX I is simultaneously synthesized from mtDNA and inserted into the mitochondrial inner membrane. Early membrane insertion is important as this subunit is highly hydrophobic, with 12 transmembrane helices, and the process is likely guided by chaperones COX14 and COX25/COA3 [30,31,32,33]. CMC1 has also been shown to stabilize this sub-assembly [34]. Before COX I can mature, three moieties must be inserted into the subunit, two hemes (heme *a* and heme *a*_3_) and one copper center (Cu_B_). COX11, a copper chaperone required for COX subunit I maturation [35], is a membrane anchored protein with an exposed copper binding site facing the IMS, allowing it to receive copper from COX17, a twin CX_9_C protein [10]. 

#### 2.1.1. COX17

COX17 and COX19 are involved in copper transport to the copper insertion chaperones during COX I assembly and are well studied in both yeast and mammals. COX17 has been extensively studied both functionally and structurally. It was first studied in *S. cerevisiae*, where it was identified in a screen for proteins that affect OXPHOS and was later shown to be a metallochaperone that localized to the cytosol and IMS [10,16,36]. However, a subsequent study showed that cycling from the cytosol to the IMS was unnecessary for COX17 function [37]. This was done by creating a SCO2/COX17 fusion protein such that the domain of SCO2 that interacts with copper was replaced by COX17 [37]. SCO2 is an inner membrane-bound protein facing the IMS that is involved in COX I assembly. IM tethered COX17 protein was found to still be able to bind copper and participate in COX biogenesis, suggesting that its copper transport function is solely within the mitochondria. COX17 functions as a copper ion transporter in the IMS, carrying copper ions to SCO1/SCO2 and COX11, which are critical assembly factors with the role of delivering copper to the redox-active metal center Cu_A_ that contains two copper ions and the single copper Cu_B_ site of the COX catalytic centers [10,38,39,40]. In fact, respiratory-deficient yeast containing a COX17 mutant are unable to grow on non-fermentable substrates unless supplemented with copper salts [10]. 

**Figure 2:**
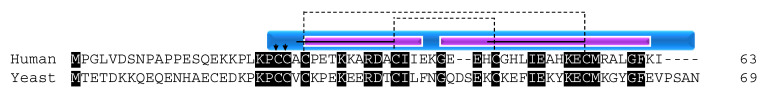
COX17. Alignment of human and yeast COX17 protein sequences. Human sequence annotation is as follows: Black boxes indicate conserved residues. Blue box indicates the CHCH domain. Purple boxes indicate a helix. Solid black lines indicate the twin CX_9_C motif. Dashed lines indicate the cysteine pairs that form structural disulfide bonds. Arrows indicate copper binding cysteines.

COX17 function is conserved across species; however, the protein sequence identity between yeast and human is only 34%. Despite this low conservation, all of the cysteines are conserved, illustrating their importance for the function of the protein (Figure 2). The cysteines of COX17 are not only important for its import, stability, and localization to the IMS, but also for its metallochaperone function. Initial functional studies in yeast determined that three cysteine residues in the CHCH domain of the protein are critical for its Cu^1+^ binding function (Figure 2) [41]. The residues are in a unique CCXC motif and, in in vitro experiments, the wildtype protein was able to bind three Cu^1+^ ions. However, when any of the cysteine residues was mutated to a serine, the result was a non-functioning COX complex, despite retaining the ability to bind copper and localize to the IMS [41]. The ability of COX17 to bind copper ions was only lost when cysteine residues 23 and 24 were mutated to serine. Evidence supporting the exact function of C26 is lacking although it is hypothesized, given its close proximity to the copper binding cysteines, to be involved in ligand transfer [41].

The evidence that COX17 could still maintain the ability to bind copper if any one of the cysteines was mutated but would still result in respiratory-deficient yeast was an interesting finding. The data suggested that more than just the CCXC motif was necessary for COX17-mediated copper transport that would result in proper functional maturation of COX. Further studies determined that COX17 exists in a dimer/tetramer equilibrium. COX17 proteins with any one of the critical CCXC motif cysteines mutated failed to form the higher molecular weight tetramer, suggesting that the ability for COX17 to form oligomers is functionally important in addition to binding copper [42]. The concentration of COX17 within the mitochondria exceeds 20 µM, suggesting that the protein exists primarily as a tetramer due to concentration dependence [42]. 

The COX17 function of copper transport for use in the copper centers of COX is conserved in mammals. Attempts to knock out COX17 in mice resulted in embryonic lethality [43]. As in yeast, the knockdown of COX17 results in defective COX activity [44] as well as decreased levels of mtDNA encoded COX subunits I and II [44,45]. In cell culture, COX17 retains its dual localization to the cytosol and the mitochondria, with protein levels enriched in the mitochondria [44]. It was originally thought that the protein is able to shuttle copper from the cytosol to the mitochondria [36]; however, functional studies suggest that the protein operates only in the mitochondria in mammals [46,47] due to the redox state of the protein as the cysteines are involved in metal binding, IMS import, and proper protein folding. In vitro, fully reduced COX17 is able to bind four copper ions, whereas partially oxidized COX17 with two disulfide bridges binds one metal ion with low specificity, and the fully oxidized form with three disulfide bonds is unable to interact with metals [46]. A dynamic NMR structural study further clarified that the functional form of COX17 resides in the IMS with the cysteines within the twin CX_9_C motif oxidized to retain its structure [47]. This oxidation state leaves the metal binding cysteines (residues 22 and 23 of the CCXC motif; Figure 2, arrows) reduced and able to bind one copper ion for the delivery to COX during biogenesis. When the protein is fully oxidized, the third disulfide bond forms between cysteines 22 and 23, effectively blocking the metal binding site [47].

COX17 may also play a role in the formation of supercomplexes [44,45]. The association with COX17 knockdown and COX depletion in supercomplexes likely hinges upon the oxidase not having the copper sites assembled in the holoenzyme, leading to degradation of the complex. HEK 293 cells with depleted levels of COX17 also display swollen mitochondria with disordered cristae [45]. Isolated mitochondria show roughly a 20% reduction in copper levels [45], which is different from yeast, despite having a conserved matrix copper pool from lower to higher eukaryotes [48,49]. However, given the swollen mitochondria, it may be that the reduced level in isolated mitochondria results from the isolation procedure.

Although COX17 alterations result in COX activity deficiency, pathogenic human mutations have yet to be identified in COX-deficient patients [50,51]. COX17 appears to be required for development, as loss of function mutations result in embryonic lethality, as observed in COX17 knockout mice. 

#### 2.1.2. COX19

**Figure 3:**
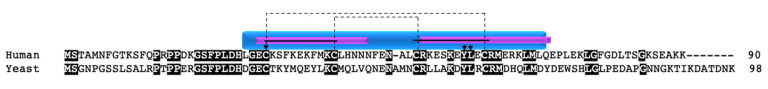
COX19. Alignment of human and yeast COX19 protein sequences. Black boxes indicate conserved residues. Blue box indicates the CHCH domain. Purple boxes indicate a helix. Solid black lines indicate the twin CX_9_C motif. Dashed lines indicate the cysteine pairs that form structural disulfide bonds. Arrows indicate copper binding cysteines.

Another well-studied copper chaperone in the twin CX_9_C family is COX19. COX19 was first identified in yeast, where knockout or mutation of the protein resulted in COX deficiency and an inability to grow on medium containing non-fermentable carbon sources [52,53,54]. COX19 retains one of the cysteines (C30) involved in the copper transport function of COX17 (C26) and localizes to the IMS and cytosol (Figure 3) [52]. Recombinant COX19 protein expressed in bacteria with copper salt supplementation purified as a monomer with copper bound [53]. In vitro experiments also showed that Cu^1+^ is bound to COX19 with a 1:1 stoichiometry and that excess amounts of Cu^1+^ resulted in a reduced spectral signal [53]. The authors suggested that, in the presence of excess Cu^1+^, there is a structural change in COX19 resulting in defective copper binding. In the same study, it was determined that both apoCOX19 and CuCOX19 exist as a dimer and that, when excess apoCOX19 is added, higher molecular weight oligomers can form. However, unlike COX17 deficiency, the addition of copper salts to growth medium does not rescue COX function and the ability for growth on non-fermentable carbon sources.

Recombinant COX19 purified directly from yeast showed that copper was bound; however, the amount bound varied across samples, which is likely due to the cysteine residues in the protein being oxidized to varying extents in vivo, although follow-up studies to confirm this hypothesis have not yet been performed [53,55]. Cysteine to alanine substitution in COX19 showed that a mutant carrying the single C30A substitution was compromised for non-fermentable carbon growth, and that the C30A/C63A double mutant was even more so. These mutants were also deficient in copper binding [53]. Although these studies show the potential for cysteine residues of COX19 to bind metal ions in vitro, it is important to perform studies regarding the redox status and potential metal binding of the protein in vivo. Until more evidence is available, it seems much more likely that the cysteine residues play a key role in the structure of the protein rather than metal binding. 

In 2015, a stable isotope labeling with amino acids in cell culture (SILAC)—mass spectrometry approach identified COX11 as a COX19 interaction partner and the apparent transient interaction was confirmed by further co-purification assays [54]. COX11 is a COX assembly factor that is important for copper insertion into COX subunit I [39,56,57,58]. When COX11 or COX19 was deleted in yeast, there was deficient COX activity and assembly [54]. Furthermore, when COX11 was knocked down, COX19 levels were depleted, suggesting that COX11 is required for COX19 accumulation or stability in the IMS. In rescue experiments, the overexpression of the proteins in opposite mutants, i.e., COX19 mutant overexpressing COX11, did not rescue the growth defect seen on non-fermentable media. The interaction of the two proteins was also shown to be dependent on the redox state of COX11, where the oxidized form of the protein has a stronger affinity for the interaction with COX19 [54]. Additionally, the same study showed that a function of COX19 is to maintain the cysteine residues at positions 208 and 210 of COX11 in an oxidized functional state through its interaction with the cysteine residue in position 111. 

In addition to the cysteine residues, yeast COX19 has a hydrophobic YL motif within each alpha helix, contained within the CX_9_C motif (CX_6_YLXC) (Figure 3, arrows) that is critical for the import of the protein into mitochondria [54]. However, only the second YL motif is conserved in humans. A COX19 mutant containing glutamates at the YL positions (COX19^EE^) was unable to localize to the IMS; even when the mutant was forced into the mitochondria via an added IMS targeting signal, it was unable to rescue COX function, remained in a reduced state, and did not interact with COX11 [54]. The defective interaction between COX19^EE^ and COX11 suggests that this hydrophobic region is important for the ability of COX19 to interact with its binding partner. 

The COX19 protein sequence is evolutionarily conserved between yeast and humans, with 35% identity. The redox-sensitive cysteines are conserved as well as one of the two YL motifs within the CX_6_YLXC motif. The difference in the YL residues between yeast and humans is potentially interesting (Figure 3, arrows). In the human protein, the YL motif is evolutionarily conserved only in the second CX_9_C motif, suggesting that the residues in the second alpha helix play a critical role in COX19 function. It would be interesting to explore whether the YL position in the human protein gives the same results when mutated to glutamates as in the above study in yeast. Despite these interesting observations, a mutational screen of 53 patients with COX deficiency in 2004 did not reveal any mutations in the COX19 gene [59]. 

As in yeast, human COX19 localizes to the mitochondria [60,61]. However, a study by Leary and others in 2012 [61] suggested an additional non-COX assembly factor role for the protein. They showed that steady-state levels of COX19 were decreased in patient fibroblasts harboring a SCO1 or SCO2 mutation, with the decrease more pronounced in the former. The cellular copper levels did not affect COX19 protein levels but did affect the localization of the protein, with the bulk of COX19 localized to the cytosol if cytosolic copper was high [61]. These findings suggest that COX19 plays a role in redox-sensitive signaling during disrupted copper homeostasis. The knockdown of COX19 in control cells resulted in approximately 40% depletion of total copper levels, and completely knocking down the protein in *SCO2* mutant cells resulted in a roughly 60% increase in copper levels [61]. A surprising finding of this study was that the knockdown of COX19 in control cells did not cause a significant change in COX activity, suggesting that the perturbation of the protein does not directly affect COX. It is possible that in humans, COX19 plays a role in SCO1-mediated signal transduction that regulates copper flux and homeostasis, rather than copper transport, which is its suggested role in yeast [61]. 

#### 2.1.3. CHCHD7/Cox23p

CHCHD7/Cox23p is a less studied protein of the family. Cox23p deletion in yeast resulted in OXPHOS deficiency with decreased levels of mitochondrial encoded subunits [62]. As with COX19, Cox23p retained one of the three cysteines found in COX17 (C26). The sequence homology of the proteins suggests that its role may be similar to that of COX17: copper transport to chaperones for maturation of COX. However, as with COX19, more protein studies are needed to support this hypothesis. OXPHOS was rescued in Cox23p-deficient yeast cells when COX17 was overexpressed in the presence of copper salts [62]. The same study showed that the proteins have similar localization to the cytosol and the IMS and that they do not interact with one another. In a screen to identify COX23 respiratory deficiency suppressors, a COX subunit I gain of function mutation was found to rescue respiration in *COX23* mutants [63]. The authors suggested that Cox23p may be involved in the biogenesis of COX I, although the exact function of the protein remains unknown.

**Figure 4:**
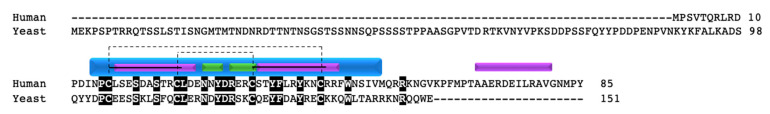
CHCHD7/Cox23p. Alignment of human and yeast CHCHD7/COX23 protein sequences. Black boxes indicate conserved residues. Human sequence annotation is as follows: Blue box indicates the CHCH domain. Purple boxes indicate a helix. Green boxes indicate a turn. Solid black lines indicate the twin CX_9_C motif. Dashed lines indicate the cysteine pairs that form structural disulfide bonds.

Cox23p is the yeast homolog of CHCHD7 and is also homologous to COX17. However, the protein identity is only 27%, with the CHCH domain conserved (Figure 4) [9,16,62]. In yeast, the CHCH domain is located at the C-terminus of the 151-residue protein, whereas in humans this domain is located at the N-terminus of the 85-residue sequence [64]. Additionally, the putative N-terminal mitochondrial targeting sequence, which is used to direct to the mitochondrial matrix, is retained in yeast but not in humans, although both proteins have been shown to localize to the IMS. Whether the targeting sequence of the yeast protein is actually needed for targeting to mitochondria remains to be tested.

Human CHCHD7 has five isoforms, some of which retain the CHCH domain [9]. Lack of the CHCH domain suggests that isoforms 3, 4, and 5 do not localize to the IMS for function in the mitochondria. Translocations and splicing events in *CHCHD7* transcripts are associated with some cancers in humans (Table 3) [65,66]. An NMR structural study showed that CHCHD7 is similar in structure to that of COX17, which contains a CHCH domain that has low hydrophobicity [64]. The study also found CHCHD7 to have high hydrophobicity in a unique, extended second alpha-helix and third alpha-helix, which is not structurally similar to COX17. However, more functional studies are required to identify the role of CHCHD7 in COX assembly. 

#### 2.1.4. CMC1

**Figure 5:**
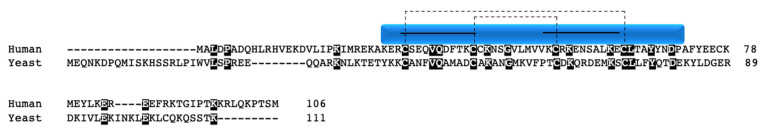
CMC1. Alignment of human and yeast CMC1 protein sequences. Black boxes indicate conserved residues. Human sequence annotation is as follows: Blue box indicates the CHCH domain. Solid black lines indicate the twin CX_9_C motif. Dashed lines indicate the cysteine pairs that form structural disulfide bonds.

CMC1 was identified in a screen seeking novel candidate proteins involved in copper metalation of COX and SOD1, as well as in copper trafficking in the mitochondria [67]. The study further revealed that CMC1 in yeast is capable of binding Cu^1+^ in a similar manner to COX17 and COX19 in vitro and that perturbation of the gene results in decreased respiration, defective COX assembly, and inability to grow on non-fermentable substrates. However, the defect could be rescued by supplementation with copper. CMC1 is localized to the mitochondrial inner membrane, facing the IMS [67]. The yeast protein has an unpaired cysteine in its C-terminal region (Figure 5). However, mutation of this cysteine does not result in disrupted mitochondrial import or a growth defect on non-fermentable substrates as compared to mutations of the CX_9_C cysteines; thus, the functional ramifications of the unpaired residue are not at present clear in yeast [68]. 

CMC1 is conserved, with a 22% sequence identity between humans and yeast (Figure 5). Human CMC1 has two unpaired cysteines: one is located directly next to the first CX_9_C motif, near the middle of the protein sequence, and the other at the N-terminal end. Human CMC1 localizes to the mitochondria and has been shown to interact directly or indirectly with COX via co-IP experiments [34,67,68,69]. CMC1 knockout in HEK 293T cells reduced the stability of COX subunit I, decreased cellular respiration, COX activity, and level of supercomplexes containing COX [34]. All of these data suggest that CMC1 plays a role in COX assembly, stability, or maturation. In the same study, COX I was shown to form an early COX assembly intermediate with CMC1, COA3, and COX14, steps that are upstream of the incorporation of subunits IV and Va and are important in the post-translational stabilization of the subunit [34]. In yeast, the expression of COX I is tightly regulated though complex IV assembly; however, in humans, this does not seem to be the case. When COX biogenesis was disrupted in various backgrounds (COX I, COX II, COX III cybrids, COX Va, and COX IV silenced, and knockout of COX20, a COX II chaperone) the CMC1-COX I-COA3-COX14 complex still formed, resulting in stabilized COX I [34]. Taken together, human CMC1 appears to be a non-essential COX I chaperone that assists in stabilizing the newly synthesized subunit in an intermediate complex with COA3 and COX14 prior to downstream COX assembly events. This suggests that mutations causing functional disruptions in CMC1 could lead to destabilization of COX I and downstream complex IV assembly. Additional studies need to be performed in order to understand the association between CMC1 mutations and human disease. 

#### 2.1.5. CMC2

CMC2 shares 50% sequence identity with CMC1, is evolutionarily conserved, and has a similar localization pattern to the inner mitochondrial membrane [70]. A CMC2-null yeast strain cannot grow on non-fermentable carbon sources and cannot be rescued by overexpression of CMC1; it has a very low level of cellular respiration, depleted COX activity, and lower levels of complex subunits, with COX subunits I and II being undetectable [70]. The study also showed that production of COX I was severely diminished in CMC2-null yeast cells, suggesting that the perturbation of the protein slows down mitochondrial translation of the core subunits due to deficient COX assembly, as has generally been reported for mutations in COX assembly proteins [30]. A portion of CMC2 was shown to co-sediment and co-IP with CMC1, and additionally, CMC1 levels were increased in the CMC2 KO strain [70]. It is thus clear that the two proteins interact in some capacity and that, in the absence of CMC2, CMC1 may be upregulated or stabilized as a compensatory mechanism. However, unlike CMC1 mutant strains, when CMC2-null yeast cells are supplemented with copper salts, they are still unable to grow on non-fermentable carbon sources, even in the presence of overexpressed CMC1, COX11, or SCO1 [70]. These data suggest that CMC1 and CMC2 perform non-overlapping but cooperative functions in the trafficking of copper for use in COX biogenesis in yeast. 

**Figure 6:**
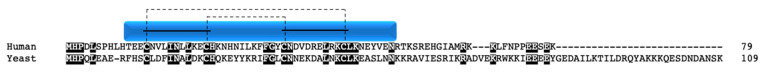
CMC2. Alignment of human and yeast CMC2 protein sequences. Black boxes indicate conserved residues. Human sequence annotation is as follows: Blue box indicates the CHCH domain. Solid black lines indicate the twin CX_9_C motif. Dashed lines indicate the cysteine pairs that form structural disulfide bonds.

As noted above, CMC2 is evolutionarily conserved from yeast to humans, with 32% sequence identity (Figure 6), although the human form of the protein is smaller than the yeast homolog. As in yeast, human CMC2 shares considerable sequence overlap with human CMC1, with cysteines in the corresponding positions and similar localization to the mitochondria in a cell culture system [70]. Although one could suggest that the human protein performs a similar function as its yeast homolog, the example of COX19 serves as a reminder that this may not necessarily be true until proven experimentally. Because such data are lacking in humans with regard to CMC2 and its function in COX assembly, a number of questions remain unanswered. 

#### 2.1.6. COA5/Pet191p

**Figure 7:**
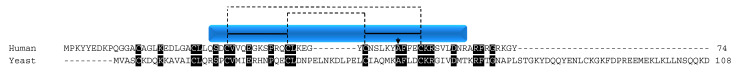
COA5/Pet191p. Alignment of human COA5 and yeast Pet191p protein sequences. Black boxes indicate conserved residues. Human sequence annotation is as follows: Blue box indicates the CHCH domain. Solid black lines indicate the CX_9_C-CX_10_C motif. Dashed lines indicate the cysteine pairs that form structural disulfide bonds.

Pet191p was first studied in yeast [71,72], and later an ortholog was identified in humans (C2orf64; COA5). The sequences between species have 32% sequence identity (Figure 7). Instead of having a true twin CX_9_C motif, the protein has a CX_10_C-CX_9_C motif. Pet191p was shown to localize to the mitochondrial IMS side of the IM in yeast [73]. In *PET191* mutant yeast, COX activity was completely diminished, as was the ability of the mutant strain to grow on non-fermentable media [72,73]. Cysteine to alanine mutational studies indicate that most of the cysteines are of some importance to facilitate growth on non-fermentable media, including C5 and C56, where no growth was seen, and C15, C32, and C46, where growth was partially inhibited [73]. As with all twin CX_9_C family members, this suggests that the cysteines are important for the formation of disulfide bonds (Figure 7). Additionally, the dual expression of HA- and Myc-tagged Pet191p with pulldown from mitochondrial lysates showed that the protein interacts with itself since both tags were present in the bound fraction [73]. 

In humans, COA5 has been implicated in early COX assembly, where 2D-blue native gel analysis shows the protein in an intermediate step containing COX I, but not the other core subunits or subunits Vb or IV [74]. In the same study, a rare mutation in COA5 was shown to be associated with hypertrophic cardiomyopathy, where both patients (siblings) died shortly after birth. Both patients were homozygous for the A53P mutation discovered in the protein (Figure 7, arrow). Cultured fibroblasts from each patient showed COX deficiency both in activity and levels of both mitochondrial and nuclear encoded subunits [74]. When the patient fibroblasts harboring the A53P mutation were infected with a retrovirus expressing WT COA5, COX subunit levels were restored. Although the exact mechanism of COA5 in COX I assembly has yet to be shown, this study does suggest that mutations within the CX_9_C motif can interfere with protein function, likely through misfolding, as postulated by the authors. 

### 2.2. COX Subunit II Module Assembly

COX II assembly is another important module in COX complex assembly (Figure 1). Copper insertion into the Cu_A_ site, which is required for the maturation of COX II, has been shown to take place in the mitochondrial IMS [19,75]. COX17 has also been shown to deliver copper to the binuclear Cu_A_ site, which is facilitated by the transfer of the ion to SCO2 via SCO1 [24,76]. 

#### COA6

**Figure 8:**
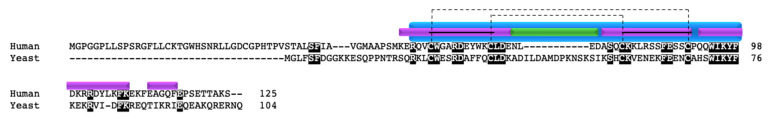
COA6. Alignment of human and yeast COA6 protein sequences. Purple boxes indicate a helix. Black boxes indicate conserved residues. Human sequence annotation is as follows: Blue box indicates the CHCH domain. Green boxes indicate a turn. Solid black lines indicate the CX_9_C-CX_10_C motif. Dashed lines indicate the cysteine pairs that form structural disulfide bonds.

COA6 is not a classic twin CX_9_C family member as it contains a single CX_9_C motif and a CX_10_C motif (Figure 8). It was first described in yeast as a novel Mia40 substrate and COX assembly factor [29]. When COA6 was deleted, yeast colonies were unable to grow under aerobic conditions, mitochondrial encoded COX subunit levels were decreased compared to WT, and overall complex IV levels were reduced in supercomplexes [29,77]. Copper supplementation [77] or treatment with elesclomal (an anticancer drug that can mimic a copper metallochaperone) [78] were able to rescue COX assembly in COA6 knockout yeast, suggesting that the protein is involved in the assembly of the copper sites either directly or indirectly. 

Numerous studies have shown that COA6 and newly synthesized COX II interact with one another [79,80]. Pathogenic mutations of COA6 resulted in abrogation of this interaction [79,81], possibly due to the mislocalization of the protein within the mitochondria [79] or misfolding of the protein [80]. COA6 was also shown to interact with the copper insertion machinery for COX II, specifically the SCO1 [80] and SCO2 [79] proteins. A later study provided insight into the interaction with the SCO proteins, showing that COA6 preferentially interacts with SCO1 in the presence of both SCO proteins [82]. Additionally, the levels of mitochondrial COA6 are dependent on copper availability in yeast [81], further supporting its role in the copper chaperone machinery. In addition to interacting with SCO2, COA6 was also shown to interact with COX12/COX VIb and all three of the proteins had overlapping but non-redundant roles in COX II maturation [81]. Interestingly, purified recombinant COA6 is able to bind copper [79,80] despite its lack of the CXCC motif found in other copper binding twin CX_9_C proteins. Structural studies have shown that recombinant WT COA6 can bind Cu^1+^ at a 1:1 stoichiometry and that the binding interface is dependent upon cysteines C58 and C90 [83]. This is likely because COA6 was shown to exist within the mitochondria in a partially oxidized state [80]. However, later studies determined that the binding of copper by COA6 may not be physiological [82], instead showing that COA6 acts as a thiol-disulfide reductase when interacting with SCO1, SCO2, and COX II [82,84]. The thiol-disulfide reductase activity of the protein would allow the cysteines of SCO1, SCO2, and COX II to be modified as the copper is passed along from chaperone to chaperone until finally reaching its site in COX subunit II.

In humans, missense mutations in COA6 were identified in two unrelated patients who died from neonatal hypertrophic cardiomyopathy with reduced COX activity in the heart [80,85,86]. One patient had a compound heterozygous mutation, resulting in amino acid changes that reside in the CX_9_C-CX_10_C domain of the protein: W59C and E87X (truncation) [85]. The missense mutation at position 59 creates an extra cysteine within the CX_9_C motif and the nonsense mutation in the CX_10_C motif results in truncation and loss of a critical domain for mitochondrial import. The second patient, who also displayed muscle hypotonia and lactic acidosis, harbored a homozygous missense mutation resulting in a W66R change shown to cause decreased COX activity in fibroblasts [86]. The human point mutations were unable to fully rescue COX deficiency in COA6 knockout yeast strains, suggesting that the mutations are pathogenic, perhaps by disrupting COA6 import into the IMS [77] or protein–protein interactions necessary for its function [82]. Additionally, the crystal structure of W59C displayed differences compared to WT, including oligomerization of the mutant and differences in charge distribution, which may account for the changes seen in protein function [83]. 

In order to understand the importance of COA6 in heart development, a study in zebrafish embryos looked for cardiac defects in a COA6 depleted background [77]. Indeed, pericardiac edema and reduced heart rate were observed in COA6 knockdown embryos, as were reduced COX II levels. In mammalian cell culture, the knockdown of COA6 resulted in reduced oxygen consumption and COX activity, supporting its role in COX biogenesis in mammals [79,80,84]. Interestingly, exogenous copper supplementation in mammalian cells does not rescue COA6 deficiency [86] as it does in yeast, suggesting a more complex mechanism in the mammalian system.

### 2.3. Undetermined

#### CHCHD8/COA4

**Figure 9:**
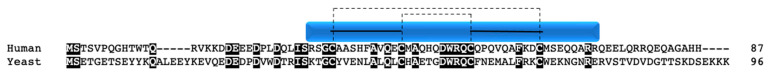
COA4/CHCHD8. Alignment of human and yeast COA4 protein sequences. Black boxes indicate conserved residues. Human sequence annotation is as follows: Blue box indicates the CHCH domain. Solid black lines indicate the twin CX_9_C motif. Dashed lines indicate the cysteine pairs that form structural disulfide bonds.

COA4 is conserved from yeast to humans (Figure 9). It is an inner membrane-associated protein facing the IMS that was shown to affect COX assembly in yeast but the overexpression of cytochrome *c* rescued COX activity defects of COA4 mutant cells [28]. The findings of the study did not reveal a direct interaction of COA4 and COX but instead suggested that COA4 helps to stabilize the membrane association of cytochrome *c* in these cells. This agrees with other studies showing that cytochrome *c* is implicated in the assembly and stability of COX [87,88]. A later study in yeast confirmed that knockout of COA4 results in a reduced oxygen consumption rate, reduced COX activity, reduced levels of the COX core subunits, and increased hydrogen peroxide production [89]. More studies are required, however, including in the mammalian system, to better understand the role of COA4 in COX assembly.

## 3. COX Structural Subunits

### COX VIb1/Cox12p

**Figure 10:**
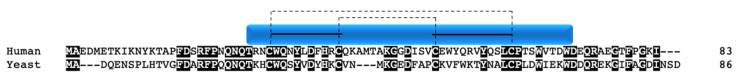
COX VIb1/Cox12p. Alignment of human and yeast COX VIb1/Cox12p protein sequences. Black boxes indicate conserved residues. Human sequence annotation is as follows: Blue box indicates the CHCH domain. Solid black lines indicate the CX_9_C-CX_10_C motif. Dashed lines indicate the cysteine pairs that form structural disulfide bonds.

Although not a true twin CX_9_C protein, this protein is often considered an “unofficial” family member in the context of COX due to structural similarity and its role in complex IV function. The nuclear encoded Cox12p/COX VIb1 has one CX_9_C motif and one CX_10_C motif. Cox12p in yeast was first shown to be a novel subunit that is required for proper COX activity but not complex assembly [90]. Cox12p protein is associated specifically with COX II biogenesis, where interaction studies show its physical interaction with COX II, COA6, and SCO1/2 [81]. The *COX6B* gene was mapped in human heart and three pseudogenes were identified and subsequently characterized [91,92]. *COX6B1* is ubiquitously expressed across tissues whereas its isoform, *COX6B2*, is testes-specific [93]. COX VIb1 shares 45% sequence identity with Cox12p (Figure 10). In 1997, the dimeric complex of COX was crystallized from bovine heart and its structure determination confirmed that COX VIb1 is part of the COX complex, sitting on top of the dimer and seemingly forming a bridge on the IMS side of COX that allows the COX monomers to interact [2]. The crystal structure shows that COX VIb1 interacts with core subunits II and III [2] and, based on computer modeling [94], is part of the cytochrome *c* binding site of COX.

Some mutations in *COX6B1* have been associated with COX deficiency disease. Two individuals with early-onset leukodystrophic encephalopathy, myopathy, and growth retardation carried a homozygous mutation in *COX6B1* resulting in an amino acid residue replacement of arginine to histidine at the N-terminus of the protein [95]. Another missense mutation, resulting in an arginine to cysteine change at the same position, was mapped in an individual with encephalomyopathy, hydrocephaly, and cardiomyopathy [96]. 

## 4. COX Regulation through Direct Interaction

### 4.1. MNRR1 (CHCHD2)/Mix17p

MNRR1 and its isoform CHCHD10 (see next section) have a common ancestor in yeast called Mix17p (formerly Mic17p), which was initially shown to localize to the nucleus [97]. However, as interest in IMS protein import increased, it was determined that Mix17p also localized to the IMS via the Mia40 pathway [98]. In a screen looking to determine which CX_9_C proteins affect OXPHOS in yeast, the knockdown of Mix17p resulted in the reduction of oxygen consumption to approximately 50% of WT [16]. The protein was later shown to be stress-sensitive using agents that induce DNA replication distress, a stimulus that also led to the characterization of changes in protein localization [99]. 

**Figure 11:**
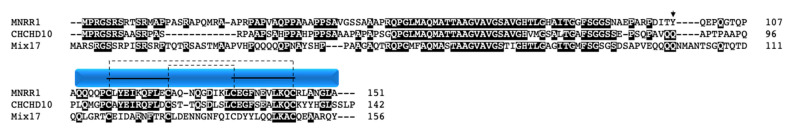
MNRR1, CHCHD10, and Mix17. Alignment of human MNRR1 and CHCHD10 (D10) and yeast Mix17 protein sequences. Black boxes indicate conserved residues. Human sequence annotation is as follows: Blue box indicates the CHCH domain. Solid black lines indicate the twin CX_9_C motif. Dashed lines indicate the cysteine pairs that form structural disulfide bonds. The arrow indicates a residue of interest discussed in this review.

Mix17p and MNRR1 have 36% sequence identity, with a centrally located hydrophobic domain, which is largely conserved (Figure 11). This overlap suggests that the hydrophobic central domain plays an important role in the function of the protein. Additionally, the cysteines in the CHCH domain are conserved in a twin CX_9_C motif. As in yeast, mammalian MNRR1 was determined to affect OXPHOS in a computational screen coupled with functional assays in human cell lines [100]. MNRR1 is also a stress-sensitive protein and displays dual localization to the mitochondria and the nucleus [101,102]. During 20% oxygen tension growth in cell culture, most cellular MNRR1 is in the mitochondria; however, at more physiological 4% oxygen tension, MNRR1 levels rapidly turn over in the mitochondria and increase in the nucleus [102].

In the mitochondria, MNRR1 has been shown to interact with COX and regulate its activity [102,103]. MNRR1 knockdown in cells affected multiple mitochondrial processes including a ~50% reduction of oxygen consumption rate, an increase in reactive oxygen species (ROS), a reduction in mitochondrial membrane potential, slower growth [102], and fragmented mitochondria, which increasingly form during oxidative stress [104,105,106]. A large-scale protein–protein interaction study showed that MNRR1 interacts with two COX subunits on the IMS-facing side of the IM (COX VIc and COX VIa1), as well as cytochrome *c*, further suggesting that MNRR1 is a regulator of COX activity [107]. However, further work will be needed as the interaction study, which was carried out with total cell lysate, also identified an interaction with wholly matrix-localized COX subunits yet MNRR1 has not been detected in the matrix [108]. 

The interaction between MNRR1 and COX is promoted when MNRR1 is phosphorylated at tyrosine residue 99 (Figure 11, arrow) by Abl2/Arg kinase [103]. When Y99 is mutated to glutamate to mimic phosphorylation, an increase in oxygen consumption is detected compared to WT. MNRR1 has been shown to have another phosphorylation site, in the retained (non-cleaved due to IMS localization) putative mitochondrial targeting sequence of the protein. 

Serine residue 41 has been shown to be phosphorylated in breast cancer and levels of MNRR1 are also increased with tumor grade, seemingly aiding the aggressiveness of breast cancer cells [109,110]. Studies have shown that oxygen consumption increases with rising levels of MNRR1, as does tumor grade [109]. MNRR1 is also associated with lung and liver cancer [111,112], and the neurodegenerative disorders Parkinson’s disease (PD) [113], Huntington’s disease [114], and lissencephaly (a neuronal migration disorder) [115]. MNRR1 has been shown to regulate cell migration [116], suggesting that it could be important for metastasis in cancer and affecting migration in lissencephaly. However, in most cases, the mechanistic involvement of MNRR1 in the disease is unknown. Of the disease-associated mutations of MNRR1, T61I (PD) is perhaps the best studied. In HEK 293 cells, overexpression of the T61I mutation results in a greater binding affinity for endogenous CHCHD10 and endogenous MNRR1 interaction [117]. However, it is important to note that the presence of increased CHCHD10 in the pulldown performed could be due to the protein binding to the endogenous WT MNRR1 in the pulldown, and not necessarily due to interaction with the overexpressed mutant. This would suggest an alteration in MNRR1 function in the mitochondria, including COX regulation.

In the nucleus, MNRR1 acts as a transcriptional activator by binding to RBPJκ at the highly conserved oxygen responsive element (ORE) in the *COX4I2* promoter, stimulating the expression of the COX IV-2 subunit isoform, which is highly expressed in the lung, trachea, and carotid body [101,102,118,119,120]. Expression of COX IV-2 is maximal at 4% oxygen tension [101,102,118,119]. When COX IV-2 is present in the complex isolated from lung, COX activity was twofold higher than the activity of COX isolated from liver with only COX IV-1 present [121]. Other COX subunits and chaperones contain the ORE within their gene promoters, including COX VIIa2 and COA4, as well as other proteins discussed in this review (Table 4). Based on this information, MNRR1 regulates COX activity with a bi-organellar approach: both through physical interaction with the complex in the mitochondria and through stress-induced transcription in the nucleus. 

MNRR1 and CHCHD10 are the only members of the twin CX_9_C family of proteins shown to dually localize to the mitochondria and the nucleus. Interestingly, CMC1 and CMC2 also have putative nuclear localization signals on the C-terminal end of the proteins; however, these putative signals have yet to be tested by mutations and, for CMC1 and CMC2, it has yet to be shown that they localize to the nucleus. Additionally, the structures of CMC1 and CMC2 are unknown, which makes it difficult to determine if the putative NLS would be structurally accessible for nuclear localization.

### 4.2. CHCHD10/Mix17p

**Figure 12:**
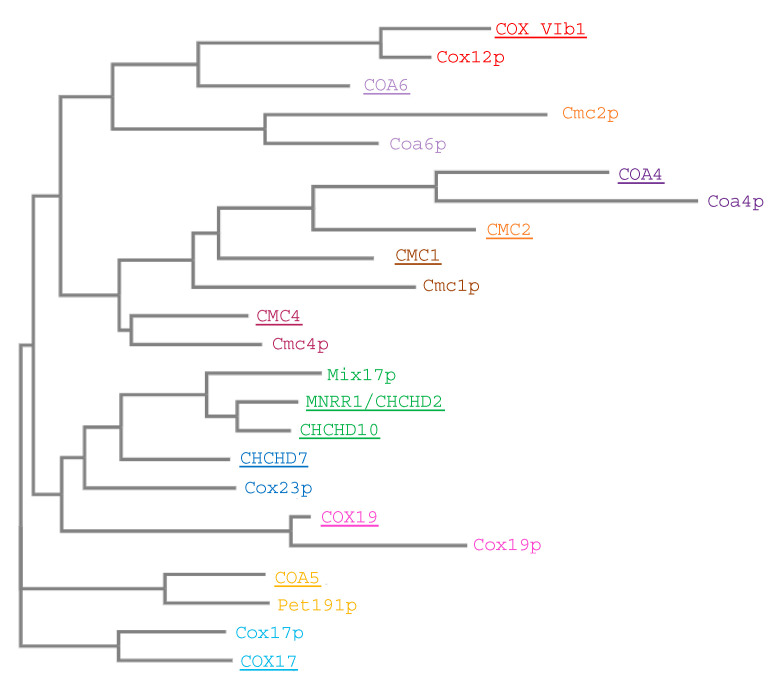
Sequence similarity of twin CX_9_C proteins. Human and yeast protein sequences were aligned using MUSCLE, phylogenetic tree created with PhyML and TreeDeign (“One Click” MAB LIRMM). Homologs/orthologs between species are colored the same. Human proteins are underlined.

Mix17p and CHCHD10 have a 33% sequence identity (Figure 11) and, like MNRR1, the central hydrophobic domain and CHCH domain are largely conserved between the two proteins. Due to the sequence homology between MNRR1 and CHCHD10 (56%) and recent common ancestor divergence (Figure 12), it was suggested that the two proteins perform similar roles within the cell [9]. Further studies provided evidence that this hypothesis was true in the mitochondria but not in the nucleus. 

CHCHD10 has been studied in the mitochondria and the nucleus [122,123,124]. Like MNRR1, CHCHD10 also localizes to the nucleus; however, at least for the *COX4I2* promoter, the protein acts as a transcriptional repressor at the ORE by binding to transcription factor CXXC5 at 8% oxygen tension in cell culture [124]. It is possible that the accumulation of MNRR1 in the nucleus at 4% oxygen displaces CXXC5 and CHCHD10 to activate ORE-mediated transcription; however, additional work is needed to characterize the mechanism in more detail. In the mitochondria, CHCHD10 localizes to the IMS and its knockdown results in decreased COX activity and ATP levels [122,125]. Like MNRR1, CHCHD10 co-purifies with COX at sub-stochiometric levels, suggesting an interaction with the complex. Experimental evidence supports an indirect interaction through MNRR1 [124]. MNRR1 knockout results in defective CHCHD10-COX binding as well as decreased COX activity [124]. In the same study, knockdown of CHCHD10 resulted in less phosphorylation of MNRR1, suggesting that CHCHD10 regulates COX through the recruitment of Arg/Abl2 kinase for MNRR1 phosphorylation. CHCHD10 has also been shown to interact with COX subunits (COX Va, COX VIc, COX VIa1, COX VIIa2) and COX assembly factor COA3 [107]. 

Much like MNRR1, CHCHD10 is associated with various neurodegenerative diseases: spinal muscular atrophy (Jokela type) (SMAJ) [126], amyotrophic lateral sclerosis (ALS) and/or frontotemporal dementia (FTD) [125,127], lower motor neuron syndrome [128], and autosomal dominant mitochondrial myopathy [123]. In all these cases, specific mutations in CHCHD10 are associated with each disease. Missense mutations S59L, P34S, and P80L are found in patients with FTD and/or ALS. Double missense mutation R15S and G58R is associated with autosomal dominant mitochondrial myopathy. Missense mutations R15L and G66V are associated with lower motor neuron syndrome and G66V is also associated with SMAJ and Charcot-Marie-Tooth disease type 2 [129]. A recent paper showed that both G66V and P80L were functionally defective in both the nucleus and the mitochondria, thereby characterizing the pathogenicity of the two mutations [124]. 

## 5. CX_9_C Proteins of Unknown Function

### CMC4

**Figure 13:**
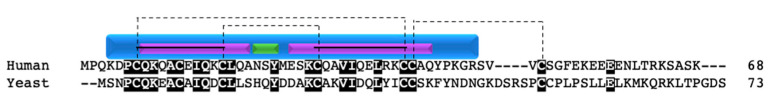
CMC4. Alignment of human and yeast CMC4 protein sequences. Black boxes indicate conserved residues. Human sequence annotation is as follows: Purple boxes indicate a helix. Green boxes indicate a turn. Blue box indicates the CHCH domain. Solid black lines indicate the twin CX_9_C motif. Dashed lines indicate the cysteine pairs that form structural disulfide bonds.

CMC4 is a CX9C family member that localizes to the mitochondrial IMS [16,98]. In yeast, CMC4 has eight cysteine residues, four of which comprise the twin CX_9_C motif. Seven of the eight cysteines are conserved in the human homolog (Figure 13). The overall sequence identity is 30%, with the yeast protein being slightly larger than the human. The limited studies in yeast show that CMC4 knockdown produces no defect in respiration [16]. However, an affinity purification–mass spectrometry proximity labeling study determined that CMC4 interacts with SCO1 in human cell cultures [130]. This suggests that CMC4 plays a non-essential role in COX II sub-assembly. One hypothesis is that CMC4 has a redundant function with another SCO1 interacting protein and somehow compensates when the other assembly factor is dysfunctional. CMC4 is associated with two diseases: T cell leukemia with translocation (X:14), where overexpression of the protein is observed [131,132,133,134,135], and frataxin mRNA deficiency [136].

## 6. Concluding Remarks

The evolutionary conservation from yeast to human of the twin CX_9_C proteins indicates that the proteins play important and conserved roles (Figure 12). This family of proteins has recently piqued the interest of investigators in the field of mitochondrial biology because (a) many family members were until recently still classified as “orphan” proteins of unknown function, and (b) the limited functional studies showed that they play important roles in the regulation of oxidative phosphorylation, seemingly through their activities relating to COX regulation. Although some of the family members are better studied than others, much remains unknown about the functional aspects of the proteins, including their underlying roles in disease. 

Over the years, many diseases have been studied that are associated with defective COX biogenesis, which often involve brain, heart, and skeletal muscle (extensively reviewed in [137,138,139]). Most of these diseases involve assembly factors, chaperones, and other regulatory proteins because COX biogenesis and activity are tightly regulated processes [140]. Mutations in SCO1/SCO2 [22,141,142,143,144], COX10 [145,146], COX15 [147], and SURF1 [21,143,148,149] have all been associated with COX dysfunction. Studies have also shown that mutations in members of the twin CX_9_C family can affect COX activity and are associated with various diseases including neurological disorders, cancers, and cardiomyopathy (Table 3). However, in most cases, the exact molecular pathogenic mechanism causing COX deficiency is unknown. Understanding the function of these proteins and the mutations that result in functional defects is critical to understanding disease etiology, which remains unknown in most cases. 

Although the proteins in this family display diverse functions, one commonality is their import into the IMS. The proteins are able to localize to the IMS through the Mia40/CHCHD4-Erv1/ALR import pathway, which relies on redox sensitivity [14,15,150,151]. An interesting consideration is that IM-bound Mia40/CHCHD4, which is also a family member and an oxidoreductase, may act as an indirect regulator of COX activity through the active or inhibited import of twin CX_9_C proteins. The IMS import machinery works through a disulfide relay system, where unfolded (reduced) twin CX_9_C proteins are brought into the IMS via the translocase of the outer membrane (TOM). The oxidized cysteine residues of Mia40/CHCHD4 then capture the reduced incoming proteins by forming disulfide bridges. Once the disulfide bonds are modified, Mia40/CHCHD4 releases the properly folded (oxidized) substrate protein into the IMS and is re-oxidized by Erv1/ALR [151]. Additionally, Erv1 has been shown to transfer electrons to Cyt*c* [152], suggesting that OXPHOS is regulated at multiple levels by the IMS import machinery.

One understudied area is that of post-translational modifications (PTMs) of the twin CX_9_C proteins. The addition or removal of PTMs affects protein activity, lifetime, and interactions. Better understanding of PTMs as regulators of COX would provide a layer of new knowledge with regard to regulation and dysregulation. A prime example is that of MNRR1, which has been shown to be phosphorylated at residue Y99 through the interaction with CHCHD10 and Abl2 kinase [103]. Phosphorylation at this site increases the activity of COX compared to WT MNRR1. However, in a family with Charcot-Marie-Tooth disease type 1A, a Q112H mutation resulted in decreased COX activity, no interaction with COX, and decreased phosphorylation of MNRR1 [103]. Another example is that of phosphorylation at serine 41, which has been associated with breast cancer in two high-throughput studies [110]. Other PTMs have also been identified for twin CX_9_C proteins although most of them derive from high-throughput studies and require functional follow-up [153]. 

In addition to enzymatic PTM additions to proteins, non-enzymatic PTMs can occur due to oxidative stress. For example, cysteine residues can be modified by reactive oxygen species. This is particularly true in the mitochondria, which are a central hub for redox signaling (see [154] for comprehensive review on cysteine-mediated redox signaling in the mitochondria). Alterations of the cysteine residues of twin CX_9_C proteins due to oxidative stress could affect IMS import, misfolding, and thereby stability and/or protein–protein interactions, and copper interaction for copper binding protein family members. In fact, tissue-specific cysteine modifications have recently been mapped in young and old mice, laying a foundational landscape upon which to study cysteine oxidation [155]. The members of the twin CX_9_C family that were annotated in this study as having cysteine oxidation are MNRR1, CHCHD7, CMC1, COA6, and COX VIb1. This is yet another area ready for investigation to further build upon the mechanistic activities of twin CX_9_C proteins during aging, stress, and disease. 

The twin CX_9_C proteins are a unique family of proteins that are emerging in the field of mitochondrial biology as important regulators of COX through assembly, structure, and direct interaction. Some of the proteins have expanded roles in mitochondrial function, such as MNRR1 and CHCHD10. These two proteins, in addition to directly regulating COX activity, also localize in the nucleus, where they function as transcription factors, modulating mitochondrial–nuclear crosstalk. In fact, many genes involved in COX biogenesis have the minimal ORE (to which MNRR1 and CHCHD10 bind) within 500 bp upstream of the transcriptional start site (Table 4). COX dysfunction is associated with numerous diseases. The “why” of COX dysfunction translating to disease is easy enough to understand: COX is the terminal enzyme of the ETC. If the complex is not working properly, the result is disrupted mitochondrial homeostasis. It is the “how” that has an important role to play in treating disease, as the assembly and regulation of the complex is finely tuned. Of course, this is a simplified way of thinking about a complex problem. Further understanding this protein family’s involvement in maintaining mitochondrial homeostasis during both normal, stress, and disease conditions will greatly add to the understanding of the regulation of COX activity and the family’s direct involvement in disease pathophysiology. 

## Figures and Tables

**Table 1:** COX regulation.

Types of Regulation of COX
Expression of tissue-, developmental-, and/or species-specific isoforms of subunits
Interaction with small molecules
Reversible phosphorylation of subunits
Protein–protein interactions
Supercomplex formation

**Table 2:** Human and yeast proteins, nomenclature, and functions of twin CX_9_C proteins with COX [9].

Human Protein	Yeast Protein	Function
CX_9_C Proteins		
COX17	Cox17p	COX copper chaperone
COX19	Cox19p	COX assembly
CMC1	Cmc1p	COX assembly
CMC2	Cmc2p	COX assembly
COA5	Pet191p	COX assembly
COA6	Coa6p	COX assembly
CHCHD7	Cox23p	COX assembly
CHCHD8	Coa4p	COX/complex III assembly/function
MNRR1/CHCHD2	Mix17p	Activity regulation
CHCHD10	Mix17p	Activity regulation
CMC4	Cmc4p	Unknown
COX VIb1	Cox12p	Subunit
Cytochrome *c* Oxidase		
COX I	Cox1p	Subunit
COX II	Cox2p	
COX III	Cox3p	
COX IV	Cox4p	
COX Va	Cox5Ap	
COX Vb	Cox5Bp	
COX VI	Cox6p	
COX VII	Cox7p	
COX VIII	COX8p	
COX IX	Cox9p	
COX XIII	Cox13p	

**Table 3:** COX-associated twin CX_9_C proteins and diseases.

Protein	Disease Association	Reference
COX17	COX17-null embryonic lethal in mice	[43]
COX19	None	
CHCHD7	Salivary gland pleiomorphic adenomas (benign)	[65]
	Lung adenocarcinoma	[66]
	Acute myeloid leukemia	[66]
CMC1	None	
CMC2	None	
COA5	Hypertrophic cardiomyopathy	[67]
COA6	Hypertrophic cardiomyopathy	[68,69]
CHCHD8	None	
MNRR1/CHCHD2	Parkinson’s disease	[70]
	Huntington’s disease	[71]
	Lissencephaly	[72]
	Breast cancer	[73]
	EGFR-positive non-small cell lung cancer	[74]
	Hepatitis B or C virus associated hepatocellular carcinoma	[75]
CHCHD10	Spinal muscular atrophy, Jokela type	[76]
	ALS and/or front temporal dementia	[77,78]
	Autosomal dominant mitochondrial myopathy	[79]
	Lower motor neuron syndrome	[80]
	Charcot-Marie-Tooth disease type 2	[81]
CMC4	T cell leukemia with translocation (X:14)	[82,83,84,85,86]
	Frataxin mRNA deficiency	[87]
COXVIb1	COX deficiency/mitochondrial encephalomyopathy	[88,89]

**Table 4:** COX subunit and assembly genes containing oxygen responsive element (ORE) within 500 bp of transcription start site. *TCCCA* is the minimal ORE element required for R1 mediated transcription. Bold and italicized bases match the classical ORE motif found in the promoter of *COX4I2*. Shaded sequences represent the non-coding strand.

ID/Sequence Window Parameters	Gene	ORE
Published ORE	*COXIVI2*	***GGACGT TCCCA CGCTGGGGCGG***
NC_000015.10:74937913-74938533	*COXVa*	TCGGCC ***TCCCA*** AAAGT***G***CTG***GG***
		CTGTAA ***TCCCA*** GCACTTTTG***GG***
NC_000002.12:97645525-97646190	*COXVb*	TCCGCC ***TCCCA C***A***C***ACA***G***TACA
		A***GAC***T***T TCCCA C***CTCCCA***GCG***T
		CCCACC ***TCCCA*** GCG***T***TATTAAA
		***G***CC*C*AC ***TCCCA*** A***G***ACT***G***T***G***GC***G***
		***G***CTTCC ***TCCCA*** GC***C***CTC***G***AGCC
NC_000012.12:120437600-120438231	*COXVIa*	TTCTGA ***TCCCA*** GA***C***C***G***CCCTAT
		CCG***C***CC ***TCCCA C***TGGCATTAAC
NC_000029.10:3560717-35651348	*COXVIb1*	***G***ATGAA ***TCCCA*** GAAACCTCTC***G***
NC_000008.11:99891909-99892550	*COXVIc*	CCGGAA ***TCCCA*** GCACTTT***G***G***G***A
		TCGTCC ***TCCCA*** AAG***TG***CT***G***G***G***A
NC_000019.10:36152390-36153000	*COXVIIa*	TTTTAC ***TCCCA*** AAGACTTTA***G***C
		***G***CTT***G***G ***TCCCA C***CGGA***G***CC***C***AA
NC_000023.11:77899014-77899593	*COXVIIb*	TACAT***T TCCCA*** AAAG***G***CCTTTA
NC_000005.10:86617391-86618131	*COXVIIc*	TTCGA***T TCCCA*** TTATCTTTA***G***T
		TACAT***T TCCCA C***AA***T***CCTT***CGG***
		TACAA***T TCCCA C***AA***T***CCAGG***G***C
		CCCAT***T TCCCA*** TCT***T***TCTTTTC
		T**A*AC***C***T TCCCA*** G***G***GCCCTC***C***TC
NC_000011.10:63974100-63974795	*COXVIIIa*	CCGGCC ***TCCCA*** AAG***TG***CT***G***G***G***A
		CTCTAA ***TCCCA*** GCACTTT***G***G***G***A
		ACGGCC ***TCCCA*** AAAGTTCTGA***G***
		CACACC ***TCCCA C***AGCCCTTG***G***C
		CC***A***T***G***A ***TCCCA*** A***GCT***TCCC***C***TC
NC_000017.11:14068795-14069649	*COX10*	CTGGGC ***TCCCA*** TTTGATA***G***A***G***A
		TCGACC ***TCCCA*** G***GCT***CAA***GCG***A
		TCGA***GT TCCCA*** G***G***A***T***CAA***GCG***A
		CTG*C*AA ***TCCCA*** GTGCTTA***G***G***G***A
		T***G***CA***G***C ***TCCCA*** GAAGTT***G***A***C***A***G***
NC_000017.11:54968279-54969164	*COX11*	T***G***TTAC ***TCCCA C***TTC***G***CTT***C***TC
NC_000012.12:50119498-50120218	*COX14*	TC***A***GCC ***TCCCA*** A***G***TA***G***CT***G***AC***G***
		CC***AC***CC ***TCCCA C***CTCA***G***CCTCC
NC_000010.11:99731958-99732640	*COX15*	None
NC_000003.12:119677203-119677899	*COX17*	None
NC_000004.12:73069629-73030150	*COX18*	C***G***C***C***T***T TCCCA CGC***G***G***CACT***GG***
		T***GAC***C***T TCCCA*** GT***C***AA***G***CCG***G***T
NC_000001.11:244834975-244835753	*COX20*	C***G***GG***G***C ***TCCCA CGC***CCACC***C***CA
NC_000007.14:43647530-4348140	*COA1*	TCCTCA ***TCCCA*** A***G***GGT***GG***TGCA
		TAGGA***T TCCCA*** GTTT***TG***T***GC***CA
		CCTT***G***C ***TCCCA*** GAGGCATTGAC
NC_000011.10:73873321-73874201	*COA4*	TC***A***GTC ***TCCCA*** AAG***TG***CT***G***G***G***A
		CT***A***TAA ***TCCCA*** GCACTTT***G***G***G***A
		TC***A***GCC ***TCCCA*** TAA***TG***CTAG***G***A
		CTGTAA ***TCCCA*** GCA***T***TTT***G***G***G***T
		CTGTAG ***TCCCA*** GCTACTCTG***G***C
NC_000001.11:234372861-234372961	*COA6*	TT***A***TT***T TCCCA*** ACTCCCCTGCC
		***G***TGACG ***TCCCA*** GAACCCATG***G***C
		CTGTAG ***TCCCA*** GCTACCT***G***G***G***A
		CCTGCC ***TCCCA*** G***G***TA***G***CT***G***G***G***A
		A***GA***TCC ***TCCCA C***CTCA***G***CCTGC
NC_000003.12:28241299-28241909	*CMC1*	***G***C***AC***AG ***TCCCA*** AAG***T***CCT***GC***A***G***
		***G***C***AC***AG ***TCCCA*** AAG***T***CCT***G***CA***G***
		CTT***C***C***T TCCCA*** G***GCT***CCTA***C***TT
		CAC*C*T***T TCCCA*** G***G***TC***GG***CATCC
NC_000016.10:80997314-80997940	*CMC2*	None
NC_000023.11:155063965-155064531	*CMC4*	TATTA***T TCCCA*** A***G***ACTTTTTTT
		TATGA***T TCCCA*** TTT***T***ATATAT***G***
		TACTC***T TCCCA*** AA***C***ATA***GG***AT***G***
NC_000009.12:133356399-133357002	*SURF1*	CCCTCA ***TCCCA*** ACTGC***G***CCCTT
		CAC***C***CG ***TCCCA*** GC***C***CC***G***CCGCC
NC_000003.12:42804144-42804699	*HIGD1A*	CAG***C***CG ***TCCCA*** GC***C***AATCAGA***G***
		ACGGAC ***TCCCA*** GC***C***CCCACCCC
NC_000007.14:10940000-10940611	*NDUFA4*	CTCG***G***C ***TCCCA*** GAGGC***G***CCGC***G***
		CTG***C***CC ***TCCCA*** GC***C***AA***GGG***TCC
NC_000007.14:56106405-56106909	*CHCHD2/MNRR1*	CCCGCC ***TCCCA*** TCT***T***CC***GG***TCT
		T***G***GTTG ***TCCCA CG***TCC***GG***AG***G***C
NC_000008.11:56212000-56212924	*CHCHD7*	CTGAT***T TCCCA*** TC***C***A***G***T***GG***TTC
NC_000022.11:23767863-237687376	*CHCHD10*	TCCCC***T TCCCA*** GCTG***G***CCC***C***AT
		***G***C***A***G***G***G ***TCCCA*** TTTCCA***G***C***CG***A
		CCCG***G***G ***TCCCA C***CGCC***G***CCACC
NC_000020.11:18137355-18138051	*PET117*	C***G***GG***GT TCCCA*** TC***C***GT***GG***AGTT
		CCC***C***TC ***TCCCA C***AGAC***G***TC***C***CT
NC_000017.11:10697489-10697998	*SCO1*	None
NC_000022:11:50524401-50524931	*SCO2*	CC***AC***AC ***TCCCA*** GCAGAAAA***C***CT
		T***G***GT***G***A ***TCCCA*** G***G***TA***G***A***GG***ACA

## Data Availability

Not applicable.
